# Complete Genome Sequence of the Type Strain Citrobacter rodentium DSM 16636

**DOI:** 10.1128/mra.01237-21

**Published:** 2022-04-05

**Authors:** Anika Wahl, Klaus Neuhaus

**Affiliations:** a Core Facility Microbiome, ZIEL-Institute for Food & Health, Technical University Munich, Freising, Germany; Indiana University, Bloomington

## Abstract

The type strain Citrobacter rodentium DSM 16636 was characterized in 1995. This species is widely used in rodents to study the virulence of locus-of-enterocyte-effacement-type pathogens, such as enterohemorrhagic Escherichia coli. The type strain had not been sequenced yet. Here, we report the closed genome (5.3 Gbp) and its plasmid (39.3 kbp).

## ANNOUNCEMENT

Citrobacter rodentium was first described in 1995 by Schauer et al. (1) as mesophilic, Gram-negative, rod-shaped enterobacterium, originally isolated from mice. Strain CDC1843-73, first classified as an atypical Citrobacter freundii strain, was later defined as the type strain, DSM 16636 (1).

C. rodentium is a natural enteric mouse pathogen and it causes transmissible murine colonic hyperplasia. Due to its similarity in patho-mechanisms to enterohemorrhagic and enteropathogenic Escherichia coli, as it also has a locus of enterocyte effacement (LEE), this species is widely used as a surrogate for human gastrointestinal diseases in mouse models. Other core virulence factors are shared as well (2–4). For in-depth analysis, complete genomes are important. However, while two other C. rodentium genomes are published (i.e., ICC 168 and DBS 100 [2, 5]), the type-strain genome was only available in fragments.

The type strain is deposited in several strain collections (e.g., DSM 16636, ATCC 51116, NBRC 105723, CIP 104675, CCUG 30795, JCM 14073, CCM 7398), including in the Weihenstephan Strain Collection as WS 4383. Here, we analyzed the strain from the Weihenstephan Strain Collection, which had been previously obtained from DSMZ (Braunschweig, Germany). C. rodentium was grown aerobically in liquid lysogeny broth at 37°C. Total genomic DNA was extracted using phenol/chloroform extraction with CTAB (cetrimonium bromide) (6). Coextracted RNA was digested with RNase A (20 mg/mL; Thermo Fisher Scientific, USA) according to the manufacturer’s protocol.

The extracted DNA (without previous shearing) was sequenced by SNPsaurus (Oregon, USA). Sequencing was performed using Pacific Biosciences (PacBio) RS II sequencing technology using one single-molecule real-time (SMRT) cell. SMRTbell libraries were made from genomic DNA (gDNA) using the Express Template prep kit 2.0 from PacBio according to the manufacturer’s protocol v2.0. Samples were pooled into a single multiplexed library and size-selected using Sage Sciences’ BluePippin system according to the manufacturer’s recommendations using the 0.75% DF marker S1 high-pass 6-kb to 10-kb v3 run protocol and S1 marker. A size selection cutoff of 8,000 bp (start value) was used. The size-selected SMRTbell library was annealed and bound according to the SMRT Link setup and sequenced on a Sequel II instrument.

Next, 45,641 raw PacBio reads were converted to fasta format with SAMtools v1.9 (7). Genome assembly was performed with Flye v2.8.3-b1695 with the following parameters: –plasmids –iterations 2 –asm-coverage 120 (8, 9); the results were assembled into a genome with a total length of 5,381,137 bp with a mean coverage of 84×, consisting of two contigs. One contig consists of the genome (*N*_50_, 5,341,875 bp; coverage, 82×; GC content, 54.63%), and the other contig is a plasmid named pCRTS (for plasmid of C. rodentium’s type strain) with 39,262 bp and a coverage of 384×. Thus, we expect about 4 to 5 plasmids per genome under the growth conditions used here. Genome annotation was performed by NCBI with PGAP v5.3 (10, 11). The prediction resulted in 4,992 coding sequences, including 22 rRNAs, 2 repeat regions, 88 tRNAs, 1 transfer-messenger RNA (tmRNA), and 4,326 unique protein-coding genes.

### Data availability.

The raw sequence reads have been deposited at the NCBI Sequence Read Archive under the SRA accession number SRR18162050 (BioProject number PRJNA759082, BioSample number SAMN21155799). The GenBank accession numbers are CP082833 (genome) and CP082834 (pCRTS). For all sequences, the first versions are described in this paper.
